# Structural characterization and comparison of three acyl-carrier-protein synthases from pathogenic bacteria

**DOI:** 10.1107/S0907444912029101

**Published:** 2012-09-13

**Authors:** Andrei S. Halavaty, Youngchang Kim, George Minasov, Ludmilla Shuvalova, Ievgeniia Dubrovska, James Winsor, Min Zhou, Olena Onopriyenko, Tatiana Skarina, Leka Papazisi, Keehwan Kwon, Scott N. Peterson, Andrzej Joachimiak, Alexei Savchenko, Wayne F. Anderson

**Affiliations:** a Center for Structural Genomics of Infectious Diseases, USA; b Department of Molecular Pharmacology and Biological Chemistry, Feinberg School of Medicine, Northwestern University, Chicago, IL 60611, USA; c Structural Biology Center, Biosciences, Argonne National Laboratory, Argonne, IL 60439, USA; d Computational Institute, University of Chicago, Chicago, IL 60637, USA; e University of Toronto, Toronto, Ontario M5G 1L6, Canada; f J. Craig Venter Institute, Rockville, MD 20850, USA

**Keywords:** acyl-carrier-protein synthase, acyl carrier protein, type II fatty-acid synthesis, inhibition, 3′,5′-adenosine diphosphate, coenzyme A

## Abstract

Some bacterial type II fatty-acid synthesis (FAS II) enzymes have been shown to be important candidates for drug discovery. The scientific and medical quest for new FAS II protein targets continues to stimulate research in this field. One of the possible additional candidates is the acyl-carrier-protein synthase (AcpS) enzyme. Its holo form post-translationally modifies the apo form of an acyl carrier protein (ACP), which assures the constant delivery of thioester intermediates to the discrete enzymes of FAS II. At the Center for Structural Genomics of Infectious Diseases (CSGID), AcpSs from *Staphylococcus aureus* (AcpS_SA_), *Vibrio cholerae* (AcpS_VC_) and *Bacillus anthracis* (AcpS_BA_) have been structurally characterized in their apo, holo and product-bound forms, respectively. The structure of AcpS_BA_ is emphasized because of the two 3′,5′-adenosine diphosphate (3′,5′-ADP) product molecules that are found in each of the three coenzyme A (CoA) binding sites of the trimeric protein. One 3′,5′-ADP is bound as the 3′,5′-ADP part of CoA in the known structures of the CoA–AcpS and 3′,5′-ADP–AcpS binary complexes. The position of the second 3′,5′-ADP has never been described before. It is in close proximity to the first 3′,5′-­ADP and the ACP-binding site. The coordination of two ADPs in AcpS_BA_ may possibly be exploited for the design of AcpS inhibitors that can block binding of both CoA and ACP.

## Abstract 2

The structural characterization of acyl-carrier-protein synthase (AcpS) from three different pathogenic microorganisms is reported. One interesting finding of the present work is a crystal artifact related to the activity of the enzyme, which fortuitously represents an opportunity for a strategy to design a potential inhibitor of a pathogenic AcpS.

## 1. Introduction

Pathogenic microorganisms, including *Staphylococcus aureus*, *Bacillus anthracis* and *Vibrio cholerae*, remain a serious threat to human life owing to the infections that they cause and their increasing resistance to current antibiotics. The type II fatty-acid synthesis (FAS II) pathway of these bacteria has attracted scientific and medical research and provides multiple targets for drug discovery, since the fatty-acid products of this enzymatic network play a vital role in the life cycle of the pathogens (Maier *et al.*, 2006 ▶). FAS II is a dissociated system which is composed of discrete monofunctional proteins that have been extensively studied in *Escherichia coli* (Magnuson *et al.*, 1993 ▶; White *et al.*, 2005 ▶). In contrast, the mammalian type I FAS (FAS I) is a single homodimeric polypeptide of ∼2500 residues (Maier *et al.*, 2006 ▶, 2008 ▶; White *et al.*, 2005 ▶; Smith *et al.*, 2003 ▶; Wakil *et al.*, 1983 ▶; Leibundgut *et al.*, 2008 ▶) and the yeast FAS I is a two-subunit protein that forms a functional megacomplex (Leibundgut *et al.*, 2007 ▶, 2008 ▶; Jenni *et al.*, 2007 ▶). Differences in activity and product distribution that arise from the structural and mechanistic details of the two pathways have been used in antibacterial drug discovery (Zhang *et al.*, 2006 ▶). However, there are contradictory data about whether FAS II of the Gram-positive bacteria may be an effective drug target (Wang *et al.*, 2006 ▶, 2007 ▶; Zhang & Rock, 2008 ▶; Brinster *et al.*, 2009 ▶; Campbell & Cronan, 2001 ▶), since the pathogens can utilize exogenous fatty acids in human serum and their uptake can overcome inhibition (Brinster *et al.*, 2009 ▶). Further complications are that FAS I has been found in *Mycobacterium tuberculosis* (Schweizer & Hofmann, 2004 ▶) and FAS II enzymes are present in eukaryotic mitochondria (Chuman & Brody, 1989 ▶; Schneider, Brors, Burger *et al.*, 1997 ▶; Schneider, Brors, Massow *et al.*, 1997 ▶; Miinalainen *et al.*, 2003 ▶; Brody *et al.*, 1997 ▶).

Coenzyme A (CoA) and the holo form of acyl carrier protein (ACP) are key substrates of fatty-acid (Magnuson *et al.*, 1993 ▶), polyketide and nonribosomal peptide synthases (Lambalot & Walsh, 1995 ▶). Holo-ACP is also involved in tetrahydrofolate and lysine metabolism (Praphanphoj *et al.*, 2001 ▶; Nishida & Nishiyama, 2000 ▶; Donato *et al.*, 2007 ▶). In FAS II, functionally active ACP shuttles growing acyl chains between the initiation-module and elongation-cycle enzymes, which together with ACP (Zhang *et al.*, 2004 ▶) have been identified as key drug targets (Wang *et al.*, 2006 ▶; Zhang *et al.*, 2006 ▶). The active form of ACP is produced by a post-translational modification of a conserved serine residue of apo-ACP by a 4′-phosphopantetheine (P-pant) group transferred from CoA bound to a holo-(acyl-carrier-protein) synthase (AcpS) (Elovson & Vagelos, 1968 ▶; Flugel *et al.*, 2000 ▶). After the holo-ACP has transferred an acyl chain to the next enzyme in FAS II, a protein with ACP phosphodiesterase activity can hydrolyze the P-pant group from the carrier protein, thus completing the ACP-turnover cycle (Vagelos & Larrabes, 1967 ▶; Powell *et al.*, 1969 ▶; Jackowski & Rock, 1984 ▶; Fischl & Kennedy, 1990 ▶; Nakanishi *et al.*, 2001 ▶), which has been hypothesized to regulate FAS (Elovson & Vagelos, 1975 ▶; Vagelos, 1973 ▶). Accumulation of apo-ACP is toxic to *Escherichia coli* (Keating *et al.*, 1995 ▶) and the apo-ACP pool has only been detected under extended depletion of CoA in the presence of amino acids in the growth medium (Keating *et al.*, 1996 ▶). AcpS is the only factor that maintains the ACP pool in its holo form, implying a possible regulatory function of AcpS on FAS II.

AcpS belongs to the phosphopantetheine transferase (PPT) family of proteins, binds CoA and its derivatives and requires magnesium for activity (Carreras *et al.*, 1997 ▶; Lambalot *et al.*, 1996 ▶). The PPT-family proteins are divided into three different groups based on sequence motifs and structural features (Mootz *et al.*, 2001 ▶). Bacterial PPTs are 120-residue trimeric enzymes and belong to the group I PPTs (PPT I; Parris *et al.*, 2000 ▶; Chirgadze *et al.*, 2000 ▶; Dym *et al.*, 2009 ▶). *B. subtilis* Sfp (surfactin-producing protein) and human AcpS represent the group II PPTs (PPT II; Quadri *et al.*, 1998 ▶; Mofid *et al.*, 2004 ▶; Reuter *et al.*, 1999 ▶; Bunkoczi *et al.*, 2007 ▶). The two domains of monomeric PPT II are similar to the two adjacent monomers in the trimeric PPT I. The group III PPTs (PPT III) comprise transferases that are an integral part of yeast and fungal FAS I (Leibundgut *et al.*, 2008 ▶; Jenni *et al.*, 2007 ▶; Lomakin *et al.*, 2007 ▶).

AcpS represents an important link in FAS II regulation and hence is a possible drug target for combating bacterial infections. AcpS is essential for the viability of *M. smegmatis* and inhibitors of AcpS have recently been reported (Chalut *et al.*, 2006 ▶; Chu *et al.*, 2003 ▶; Gilbert *et al.*, 2004 ▶). While a number of crystal structures of bacterial AcpSs are available, the mechanism of the transferase activity and its inhibition are not entirely understood. The high-resolution X-ray structures of AcpS from *S. aureus* (AcpS_SA_), *V. cholerae* (AcpS_VC_) and *B. anthracis* (AcpS_BA_) reported here contribute to the understanding of the general structural and mechanistic details of the pathogenic AcpS-catalyzed reaction and suggest a strategy for inhibition.

## 2. Materials and methods

### 2.1. Protein cloning, expression and purification

AcpS_SA_, AcpS_VC_ and AcpS_BA_ were cloned into the pMCSG7 vector with an N-terminal six-His tag, expressed in *E. coli* BL21-CodonPlus(DE3) cells and purified using the immobilized metal-affinity chromatography technique as described previously (Gräslund *et al.*, 2008 ▶). AcpS_BA_ and AcpS_VC_ were expressed, purified and crystallized as SeMet proteins following well established procedures (Kim *et al.*, 2004 ▶). Dynamic light scattering and analytical gel filtration were used to analyze the homogeneity and the oligomerization state of the proteins.

### 2.2. Crystallization

Prior to crystallization trials, the proteins were stored in 10 m*M* Tris–HCl pH 8.3, 500 m*M* NaCl, 5 m*M* β-mercapto­ethanol (BME) (AcpS_SA_), 20 m*M* HEPES pH 8.0, 200 m*M* NaCl, 1 m*M* dithiothreitol (DTT) (AcpS_VC_) and 10 m*M* HEPES pH 7.5, 300 m*M* NaCl, 0.5 m*M* tris(2-carboxyethyl)­phosphine (TCEP) (AcpS_BA_) at 193 K. The proteins were crystallized with and without CoA using the sitting-drop vapor-diffusion technique and commercially available crystallization screens from Qiagen (Valencia, California, USA) or optimized sparse-matrix crystallization screens (University of Toronto). Crystals suitable for structure determination were obtained under the following conditions: 800 m*M* succinate pH 7.0 at 295 K for AcpS_SA_ (7.3 mg ml^−1^), 25% PEG 3350, 200 m*M* MgCl, 100 m*M* HEPES pH 7.5, 10 m*M* CoA at 295 K for AcpS_BA_ (13.8 mg ml^−1^) and 30% MPD, 100 m*M* sodium acetate pH 4.6, 20 m*M* CaCl_2_, 10 m*M* CoA at 289 K for AcpS_VC_ (55 mg ml^−1^). Cryoprotection was performed using 25% sucrose for AcpS_SA_, 5% glycerol, 5% sucrose, 5% ethylene glycol in magic solution, Paratone for AcpS_BA_ and 10% glycerol, 30% MPD, 100 m*M* sodium acetate pH 4.6, 20 m*M* CaCl_2_ for AcpS_VC_.

### 2.3. Structure determination

X-ray data were collected on the Life Science Collaborative Access Team (LS-CAT) 21-ID-F (AcpS_SA_ and AcpS_BA_) and the Structural Biology Center (SBC) 19-ID (AcpS_VC_) beamlines at the Advanced Photon Source, Argonne National Laboratory, USA. Diffraction images for the deposited structures are available at the CSGID website (http://www.csgid.org/csgid/pages/home). Data sets were processed with *HKL*-2000 (Otwinowski & Minor, 1997 ▶; AcpS_BA_) and *HKL*-3000 (Minor *et al.*, 2006 ▶; AcpS_SA_ and AcpS_VC_). Initial structure solutions for AcpS_SA_ and AcpS_BA_ were obtained using *Phaser* (McCoy *et al.*, 2007 ▶) from the *CCP*4 package (Winn *et al.*, 2011 ▶) using the *B. subtilis* AcpS structure (PDB entry 1f7l; Parris *et al.*, 2000 ▶) and the structure of AcpS_SA_ (PDB entry 3f09), respectively, as the molecular-replacement model. The models were rebuilt with *ARP*/*wARP* (Morris *et al.*, 2003 ▶). The structure of AcpS_VC_ was determined by single-wavelength anomalous dispersion (SAD) using the automated phasing and model-building option in the *HKL*-3000 suite (Minor *et al.*, 2006 ▶). Although 48 Se sites were expected, 61 Se sites were found by *SHELXD* (Sheldrick, 2008 ▶) because some N-terminal SeMet sites were found to have multiple sites. Phasing was carried out by *MLPHARE* (Otwinowski, 1991 ▶) with a final overall phasing power of 1.15 to 1.85 Å resolution and the phases were improved by density modification (*DM*; Cowtan, 1994 ▶; Supplementary Fig. 1^1^). Initial model building was performed with *Buccaneer* (Cowtan, 2006 ▶) and subsequent manual adjustments used *Coot* (Emsley & Cowtan, 2004 ▶) to complete the first model of AcpS_VC_ with a total of 3014 amino acids (out of 3096). The initial models were refined with *REFMAC* v.5.5 (Murshudov *et al.*, 2011 ▶; AcpS_SA_ and AcpS_BA_) and *phenix.refine* (Adams *et al.*, 2010 ▶; AcpS_VC_) with manual adjustments in *Coot* (Emsley & Cowtan, 2004 ▶). The quality of the final models was checked with the PDB validation server (*ADIT* validation server; http://deposit.pdb.org/validate/) and *MolProbity* (Davis *et al.*, 2007 ▶; Chen *et al.*, 2010 ▶). Data collection and refinement statistics for all three proteins are given in Table 1 ▶. The pairwise structural alignments were performed with the *DaliLite* server (Holm & Park, 2000 ▶). The structural figures were generated using *PyMOL* (DeLano, 2002 ▶) and *LigPlot* ^+^ v.1.3 (Wallace *et al.*, 1995 ▶).

## 3. Results

### 3.1. Overall tertiary and quaternary structure of bacterial AcpS

**Figure 1:**
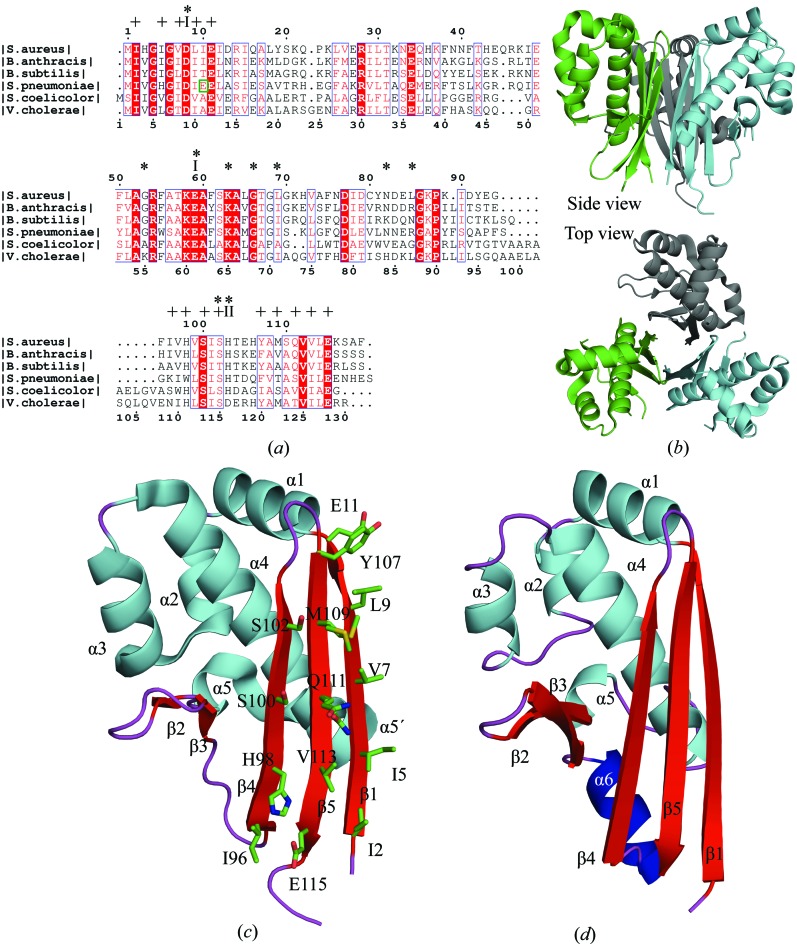
Bacterial AcpS. (*a*) Multiple sequence alignment of some bacterial AcpS enzymes with known X-ray structures: *S. aureus* (PDB entry 3f09), *B. anthracis* (PDB entry 3hyk), *B. subtilis* (PDB entry 1f7l), *S. pneumoniae* (PDB entry 1fth), *V. cholerae* (PDB entry 3qmn) and *S. coelicolor* (PDB entry 2jbz). The conserved residues are shaded red and similar residues are displayed in red letters. Residues of the central β-sheet of AcpS_SA_ numbered in (*c*) are marked with a plus sign. Asterisks indicate residues of AcpS_VC_ that coordinate CoA/K (see Fig. 3 ▶ *c*). The coordinators of two magnesium cations, Mg-I and Mg-II (see Figs. 4 ▶ *d* and 4 ▶ *e*), are shown as I and II, respectively. The green box indicates the Glu-II position, an alternative glutamate of the group II and III PPTs that is involved in coordination of Mg-I. Multiple sequence alignment was performed by *ClustalW* (available at http://www.genome.jp/tools/clustalw) and the figure was generated with *ESPript*2.2 (Gouet *et al.*, 1999 ▶). (*b*) The quaternary structure of the AcpS enzyme (apo-AcpS_SA_ is shown). (*c*) The tertiary structure of a single polypeptide of AcpS_SA_. The residues (in green) of the central β-sheet that are exposed to the interior of AcpS trimer are numbered according to the sequence of AcpS_SA_. (*d*) Monomer of AcpS_VC_ with the structurally distinct helix α6 in dark blue.

The investigated AcpS enzymes share about 50% sequence identity and all form functional trimers in the crystal with a comparable total buried surface area of ∼4500–5060 Å^2^ (Figs. 1 ▶ *a* and 1 ▶ *b*). Dynamic light scattering and analytical size-exclusion chromatography (data not shown) are consistent with the crystallographic data on oligomerization. The three proteins are structurally very similar, with root-mean-square deviations (r.m.s.d.s) of 0.14–0.90 and 1.1–1.5 Å in the C^α^-atom positions of the chains within and between the three species, respectively. The AcpS monomer has an elongated elliptical shape and possesses an α/β fold. The longest helix, α4, is wrapped by a three-stranded antiparallel β-sheet, five shorter helices and two β-strands (Figs. 1 ▶ *c* and 1 ▶ *d*). Seven of the 14 residues of the β-sheet that are buried in the interior of every ApcS trimer are hydrophobic (Fig. 1 ▶ *c*). Hydrophilic interactions also contribute to trimerization. AcpS_SA_ and AcpS_BA_ lack helix α6 (residues 92–101) of AcpS_VC_ (Fig. 1 ▶ *d*).

### 3.2. Crystal structure of *S. aureus* apo-AcpS

**Figure 2:**
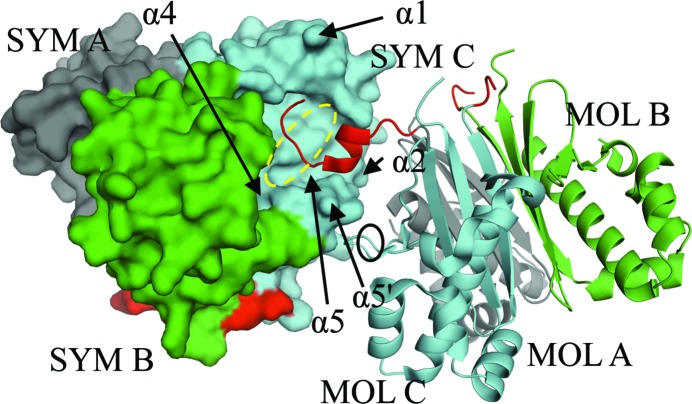
Purification tag of AcpS_SA_ in the CoA-binding site. The symmetry-related trimer is shown in surface representation. The trimer of the asymmetric unit is shown in ribbon representation. The 24-residue purification tag (red) and residues 83–84 (black oval) that stabilize the α5′-helix of SYM C are shown. A dashed yellow oval visualizes the binding area of the P-­pant portion of CoA.

13 residues in molecule *A* (MOL A), four residues in molecule *B* (MOL B) and three residues in molecule *C* (MOL C) of the 24-residue N-terminal purification tag were modeled. The purification tag of MOL A in one trimer docks onto a solvent-exposed hydrophobic patch that is formed by residues of helices α1, α2 and α4 of a symmetry-related molecule *C* (SYM C) (Fig. 2 ▶). The helical part of the tag is positioned above helix α5 and between helices α2 and α5′ of SYM C, interacting with the side chains of these structural elements (Fig. 2 ▶). The dipole moment of helix α5′ may have a weak stabilizing effect on the helical portion of the tag. Glu84 and Leu85 of MOL C contribute to the interaction by contacting helix α5′ of SYM C (Fig. 2 ▶), while both of these amino-acid residues are disordered in chain *A*. Leu85 is ordered and Glu84 is not observed in the electron density in chain *B*.

### 3.3. Crystal structure of *V. cholerae* AcpS in complex with CoA

**Figure 3:**
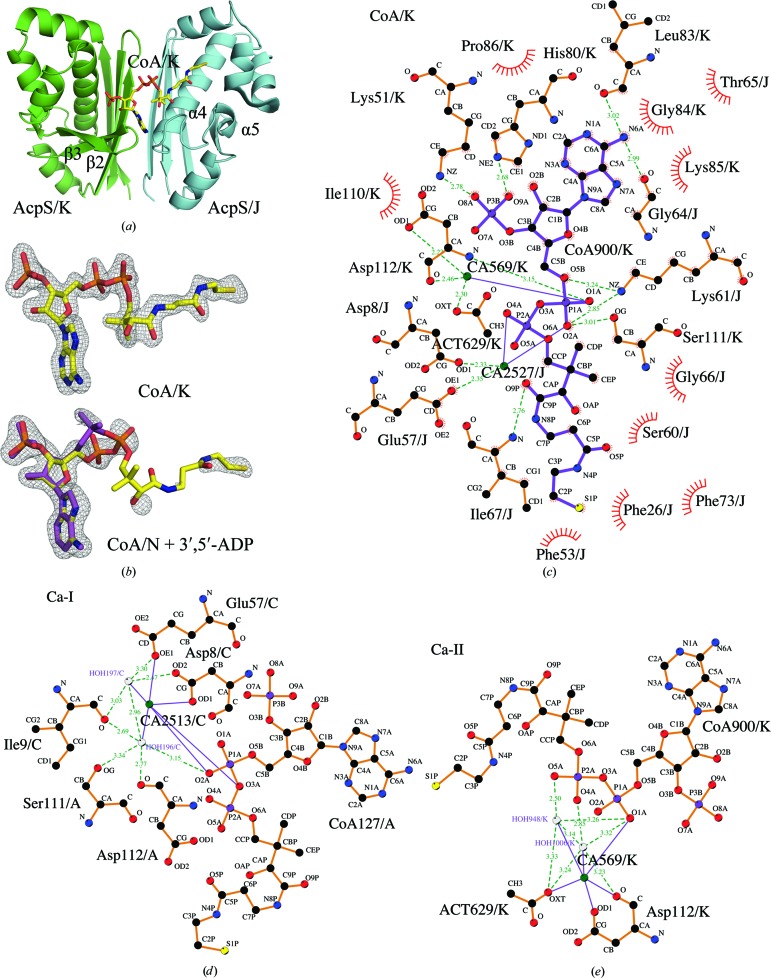
The structure of the binary CoA–AcpS_VC_ complex. (*a*) Interaction of CoA/K at the interface of the AcpS/K and AcpS/J protein molecules of the AcpS_VC_/J/K/L trimer. CoA is colored according to its atoms: C, light yellow; N, blue; O, red; P, orange; S, dark yellow. (*b*) Examples of CoA molecules with well defined (CoA/K) and poorly defined (CoA/N) electron densities. The 2*F*_o_ − *F*_c_ maps are contoured at 1.0σ. The modeled 3′,5′-ADP molecule at the CoA/N binding site is shown as purple sticks. (*c*) Coordination of CoA/K. (*d*) and (*e*) show the two calcium-binding sites, Ca-I and Ca-II. The coordination of CA2513/C in (*d*) is equivalent to that of CA2527/J in (*c*).

24 polypeptide chains of AcpS_VC_ are arranged in eight trimers in the *P*2_1_ asymmetric unit. Three molecules of CoA were modeled per trimer. Each CoA interacts with the two adjacent protein subunits in every trimer (Fig. 3 ▶ *a*). The electron density of the entire CoA is clearly defined in six of the 24 CoAs (Fig. 3 ▶ *b*). In the remaining 18 CoAs either the P-pant group has traces of density or, for the CoA P-pant group in chain *N* (CoA/N), no density was observed (Fig. 3 ▶ *b*). For this reason, a 3′,5′-ADP molecule was modeled with an occupancy of 0.2 at this binding site (Fig. 3 ▶ *b*). The pantothenate parts of all CoAs have two kinks of ∼100–110° that might be forced by intramolecular and intermolecular protein–ligand contacts.

The coordination of CoA in chain *K* (CoA/K) is described here because it has well defined density for the entire ligand (Fig. 3 ▶ *c*). The adenine ring of CoA/K is placed between the N-­terminus of strand β3 of AcpS chain *K* (AcpS/K) and the C-­terminus of helix α4 of AcpS chain *J* (AcpS/J), making interactions with Leu83 of chain *K* (Leu83/K) and Gly64 of chain *J* (Figs. 3 ▶ *a* and 3 ▶ *c*). The ribose ring does not make hydrogen bonds to the protein and its 2′-OH group points toward the hydrophobic Pro86 and Ile110 of AcpS/K (Fig. 3 ▶ *c*). The 3′-phosphate group (3′-P) interacts with the side chains of Lys51 and His80 (Fig. 3 ▶ *c*) of AcpS/K. The pyrophosphate moiety of CoA is anchored by interactions with Ser111/K and Asp112/K and with Asp8, Glu57 (through Ca^2+^) and Lys61 of AcpS/J (Fig. 3 ▶ *c*). Calcium cations with occupancies of between 0.6 and 1.0 were modeled at two different sites, Ca-I and Ca-II, in the vicinity of every CoA (Figs. 3 ▶ *d* and 3 ▶ *e*) except CoA/N. The calcium peaks were identified firstly by the presence of 20 m*M* CaCl_2_ in the crystallization condition and secondly by the coordination geometry of these peaks.

### 3.4. Unusual 3′,5′-ADP form of *B. anthracis* AcpS

**Figure 4:**
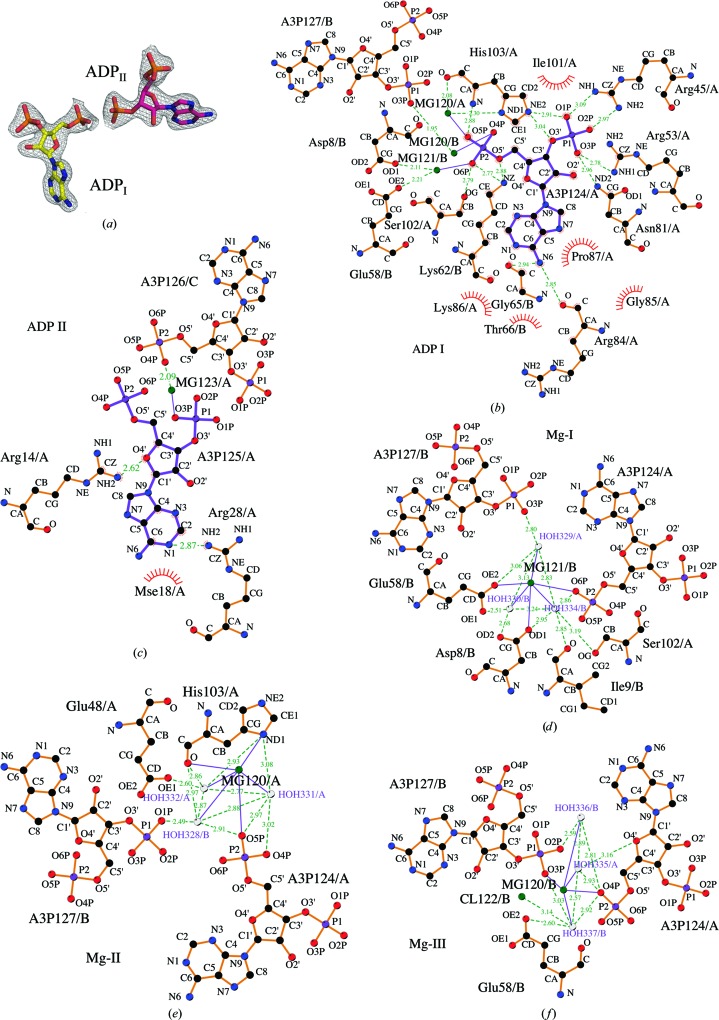
Products of CoA hydrolysis by ACP-free AcpS_BA_. (*a*) The 3.0σ OMIT map of the two 3′,5′-ADP molecules, ADP_I_ and ADP_II_, modeled at each of the three CoA-binding sites. (*b*, *c*) Binding pockets of ADP_I_ and ADP_II_. (*d*), (*e*) and (*f*) show the Mg-I, Mg-II and Mg-III binding sites.

Analysis of the initial 2*F*_o_ − *F*_c_ and *F*_o_ − *F*_c_ electron-density maps at each coenzyme-binding site of the AcpS_BA_ structure revealed the presence of two 3′,5′-ADP product molecules instead of the cocrystallized CoA (Fig. 4 ▶ *a*). Hydrolysis of CoA in the crystal or the crystallization mother liquor was possible owing to the presence of 200 m*M* MgCl_2_ in the crystallization condition. The binding of one 3′,5′-ADP molecule (ADP_I_) is similar to the adenosine diphosphate (ADP) portion of CoA in the binary CoA–AcpS complex structures [PDB entries 1f7l (Parris *et al.*, 2000 ▶), 3qmn and 2jbz (Dall’aglio *et al.*, 2011 ▶)] and of 3′,5′-ADP in the 2qg8 (Structural Genomics Consortium, unpublished work) and 1fth (Chirgadze *et al.*, 2000 ▶) structures (Fig. 4 ▶ *b*). The position of the second 3′,5′-ADP (ADP_II_) is described for the first time here (Fig. 4 ▶ *c*).

The N6 atom of the adenine ring of ADP_I_ makes hydrogen bonds to the backbone carbonyl O atoms of Arg84 of one monomer and Gly65 of the adjacent monomer in the trimer (Fig. 4 ▶ *b*). Residues Arg45, Arg53, Asn81 and His103 surround the 3′-P group of every ADP_I_, while residues Asp8, Glu58, Lys62, Ser102 and His103 interact with the 5′-phosphate group (5′-P; Fig. 4 ▶ *b*). ADP_II_ interacts with Arg14 and Arg28 of one monomer of the trimer (Fig. 4 ▶ *c*) and Lys70, Glu71 and Arg84 of a symmetry-related trimer (not shown).

Three magnesium cations, Mg-I, Mg-II and Mg-III, were modeled around the 5′-P group of each ADP_I_ (Figs. 4 ▶ *d*, 4 ▶ *e* and 4 ▶ *f*). The identity of the magnesium peaks was identified firstly by the presence of 200 m*M* MgCl_2_ in the crystallization condition and secondly by the coordination geometry and the distances to the coordinating atoms. The positions of Mg-I and Mg-II coincide with those in the AcpS_PY_ structure (PDB entry 2qg8; Vedadi *et al.*, 2007 ▶) and those of the calcium cations in the 3qmn structure. Mg-III facilitates the ADP_I_–ADP_II_ interaction. The 5′-P of ADP_I_ and the 3′-P of ADP_II_ both interact with Mg-III, so that the distance between the O4P atom of the 5′-P and the O3P atom of the 3′-P is ∼2.8 Å (Fig. 4 ▶ *f*).

## 4. Discussion

### 4.1. Overall structural comparison of AcpS

The residues that are involved in the activity and oligomerization of bacterial AcpS are highly conserved (Figs. 1 ▶ *a*, 5 ▶ *a* and 5 ▶ *b*). The partially hydrophobic surface of the three-stranded antiparallel β-sheet is considered to be the main reason why bacterial AcpS forms trimers (Fig. 1 ▶ *b*). Polar contacts between adjacent AcpS monomers in a trimer also contribute to oligomerization. Differences between the AcpS structures lie outside the regions that are required for trimerization, are species-specific and their functional consequences, if any, remain to be investigated. The residues between strand β3 and strand β4 in the structures of AcpS_VC_, *Streptomyces coelicolor* AcpS (AcpS_SC_; PDB entries 2jca and 2jbz; Dall’aglio *et al.*, 2011 ▶) and *Plasmodium yoelii* AcpS (AcpS_PY_; PDB entries 2bdd and 2qg8; Vedadi *et al.*, 2007 ▶) form an α-­helix, while they adopt a loop conformation in the structures of AcpS_SA_, AcpS_BA_ and other AcpSs (PDB entries 1f7t and 1fte; Parris *et al.*, 2000 ▶; Chirgadze *et al.*, 2000 ▶; Figs. 1 ▶ *c*, 1 ▶ *d* and 5 ▶ *c*).

The synthases do not undergo overall structural changes upon association/dissociation of CoA and ACP; however, conformational perturbations do occur at their easily accessible binding sites and in the close vicinity. The solvent-exposed loops α1–α2, α3–α4, β2–β3 and α4–α5(α5′) and the adjacent termini of the respective helices/strands of known AcpS structures can be disordered in the absence of CoA, ACP, small molecules and crystal-packing forces. However, despite the presence of ACP in the *B. subtilis* AcpS (AcpS_BS_) structure (PDB entry 1f80; Parris *et al.*, 2000 ▶), the loop residues still exhibit high *B* factors in comparison to the central β-sheet of the synthase. Thus, the conformation of all of the aforementioned regions is linked to the presence of the AcpS substrates.

### 4.2. Binding of the substrates

The residues of the CoA-binding and ACP-binding sites are well conserved in the AcpS enzymes, which suggests a similar mode of association of the substrates and mechanism of the AcpS-catalyzed reaction (Figs. 1 ▶ *a* and 5 ▶ *a*). The AcpS trimer binds three CoA molecules and three ACP molecules. The interface between two adjacent AcpS monomers serves as a docking area for both CoA and ACP.

The apo-AcpS_SA_ structure reveals that the intact purification tag may interfere with the binding of CoA (Fig. 2 ▶). This could explain why no suitable crystals were obtained of the CoA-­complexed tagged protein. Cleavage of the tag was inefficient for AcpS_SA_, possibly owing to inaccessibility of the tobacco etch virus (TEV) cleavage site. Cocrystals of CoA and AcpS_SA_ without the purification tag were also poorly ordered. Whether the purification tag was present or absent, crystals of apo-AcpS_BA_ and apo-AcpS_VC_ only diffracted to low resolution. The structure of AcpS_VC_ provides details of the interaction of CoA with the enzyme (Fig. 3 ▶ *c*). The nucleotide portions of all CoA molecules in the 3qmn structure exhibit lower *B* factors than the P-pant group. The high thermal factors and observed conformational diversity of the P-pant moiety can be explained by the mechanism of the reaction, in which accessibility of the group is essential. This has also been reported for other AcpSs (Reuter *et al.*, 1999 ▶). AcpS_BA_ only gave well diffracting crystals when the protein had the tag removed and it was pre-incubated with CoA. However, the presence of 200 m*M* MgCl_2_ in the crystallization condition appears to have been sufficient for the AcpS to hydrolyze the 10 m*M* CoA in the crystallization mixture even in the absence of ACP. Dym *et al.* (2009 ▶) have cocrystallized *M. tuberculosis* AcpS (AcpS_MT_; PDB entry 3hqj) with CoA; however, they only modeled the 3′,5′-ADP part of the substrate and suggested that the P-pant moiety was disordered in the crystal. In contrast to all these other structures, the crystal structure of AcpS_BA_ clearly shows two 3′,5′-ADP molecules, ADP_I_ and ADP_II_, at each CoA-binding site (Fig. 4 ▶ *a*). The 3′-P group of ADP_I_, which represents the normally observed nucleotide site, in the AcpS_BA_ structure makes hydrogen bonds to Arg45, Arg53, Asn81 and His103 (Fig. 4 ▶ *b*). Moreover, the position of ADP_I_ is additionally stabilized by three magnesium cations (Figs. 4 ▶ *d*, 4 ▶ *e* and 4 ▶ *f*).

### 4.3. Mechanism of the AcpS-catalyzed reaction

The phosphopantetheinylation reaction proceeds in three steps: (i) deprotonation of the serine residue of ACP, (ii) S_N_2-­type nucleophilic attack of the deprotonated serine on the β-­phosphate of the bound CoA and (iii) protonation of the α-­phosphate of 3′,5′-ADP by a conserved lysine (Lys63 in AcpS_SA_, Lys61 in AspS_VC_ and Lys62 in AcpS_BA_; Figs. 3 ▶ *c* and 4 ▶ *b*) of AcpS (Johansson *et al.*, 2009 ▶). The initial deprotonation step is achieved either by a water molecule, as in the group I PPT family (Parris *et al.*, 2000 ▶), or by a glutamate residue, as in the group II PPT family (Bunkoczi *et al.*, 2007 ▶). The preference for one or the other mechanism is determined by the coordination pattern of the Mg^2+^ denoted Mg-I (Fig. 4 ▶ *d*). If an Mg-bound hydroxide can activate a water molecule for nucleophilic attack, AcpS can hydrolyze CoA without the carrier protein (Parris *et al.*, 2000 ▶). This might explain the absence of an intact cofactor in the AcpS_BA_ structure even though the protein was cocrystallized with CoA. Binding of Ca^2+^ at the Mg-I site in the AcpS_VC_ structure (Fig. 3 ▶ *d*) might lead to distortion of the active site and inability of the enzyme to hydrolyze CoA. However, calcium can function in catalysis: Parris *et al.* (2000 ▶) have demonstrated that the P-pant group is transferred from the CoA–AcpS_BS_ complex to ACP_BS_, although at a reduced rate, if calcium is present. The modeled 3′,5′-ADP at the CoA/N binding site of the AcpS_VC_ structure is perhaps a result of slow hydrolytic activity in the absence of ACP (Fig. 3 ▶ *b*).

Two different glutamate residues, Glu-I (conserved among all PPTs) and Glu-II (specific to the group II and III PPT) (Fig. 1 ▶ *a*), have been proposed to play a role in the deprotonation step. Substitution of Glu181 (Glu-I) caused a significant loss of human AcpS activity (Bunkoczi *et al.*, 2007 ▶). Glu-I binds Mg-I through a metal-bound water molecule in the group I PPT AcpS_MT_ (PDB entry 3hqj), but was suggested to not be involved in the deprotonation step (Dym *et al.*, 2009 ▶). Dym *et al.* (2009 ▶) also suggested an alternative deprotonation scenario in AcpS_MT_ owing to the presence of the second Mg-binding site and the specificity of the synthase. In the structures of AcpS_BA_ and AcpS_MT_ (PDB entry 3hqj) a magnesium cation is bound at this site, denoted Mg-II (Fig. 4 ▶ *e*). A calcium cation was also modeled at this site in the AcpS_VC_ structure (Fig. 3 ▶ *e*). Many Mg-binding enzymes (reviewed in Black *et al.*, 1994 ▶; Glusker *et al.*, 2001 ▶) bind two magnesium cations at a distance of 3.6–6.3 Å apart. Although one Mg^2+^ is considered to be a catalytic metal, the role of the second Mg^2+^ is unclear; it may contribute to the maintenance of structural integrity at the active site or may possibly lower the activation barrier of a reaction (Black *et al.*, 1994 ▶). Mg-I and Mg-II are ∼5 Å apart in the AcpS_BA_ and AcpS_MT_ structures. Mg-III is also a distance of ∼5 Å from Mg-I and Mg-­II and bridges ADP_I_ and ADP_II_ in the AcpS_BA_ structure. The observation of this site is likely to be a consequence of the high concentration of the metal and the presence of the hydrolyzed product 3′,5′-ADP (Fig. 4 ▶ *f*).

### 4.4. Inhibition of bacterial AcpS

**Figure 6:**
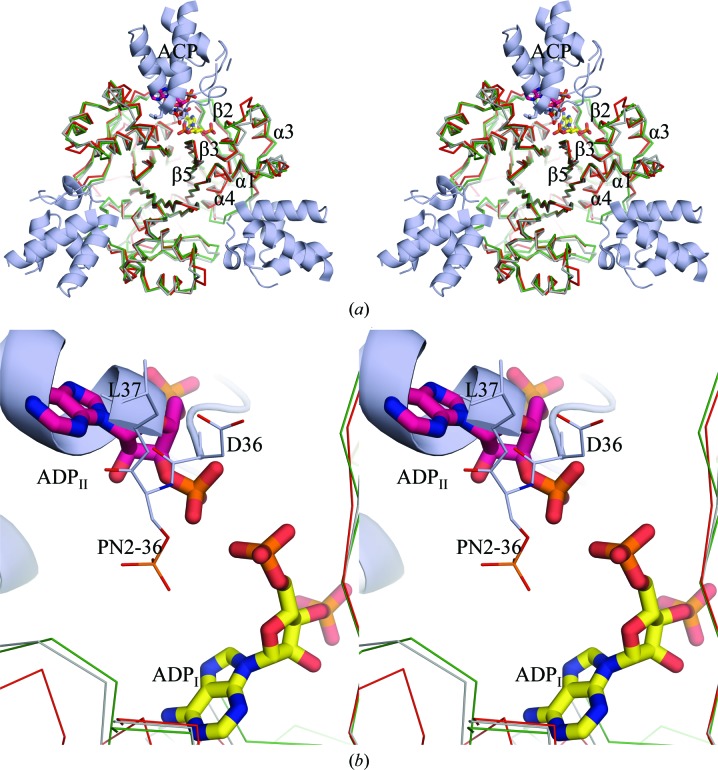
Modeling the bacterial AcpS–ACP complex. (*a*) Stereogram of the least-squares superposition of the AcpS_SA_ (red), AcpS_BA_ (green) and AcpS_BS_–ACP_BS_ (AcpS, black; ACP, light blue ribbon) structures. Two 3′,5′-ADP molecules in the *B. anthracis* structure are shown a sticks. (*b*) Stereo close-up view of (*a*) showing ADP_II_ in the AcpS_BA_ structure clashing with the α3 helix of ACP_BS_. Asp35, PN2-36 (P-pant-modified Ser36) and Leu37 of ACP are depicted as lines and colored by element: C, blue; O, red; N, dark blue; P, orange.

**Figure 7:**
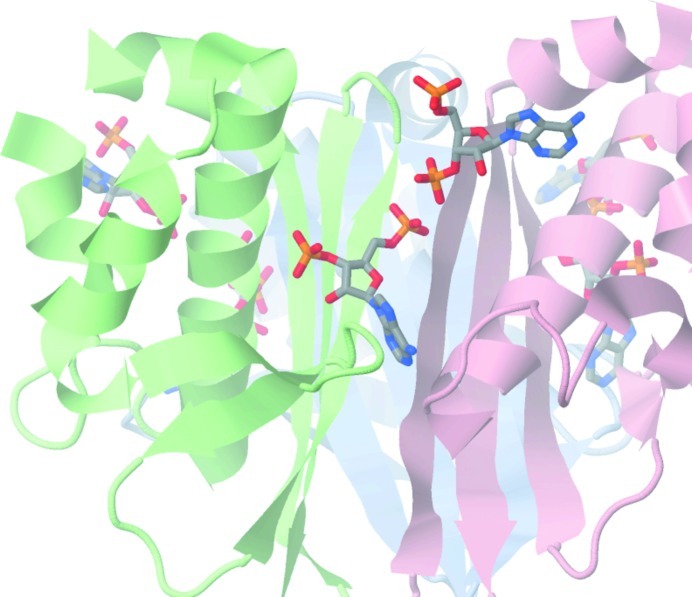
Strategy for inhibition of bacterial AcpS.

Binding of more than one ligand to other proteins has been reported and utilized in the design and optimization of inhibitors. For example, two inhibitor molecules were found in the active site of an aspartic proteinase, plasmepsin II from *Plasmodium falciparum* (PDB entry 2bju; Prade *et al.*, 2005 ▶), and two ligands bind the E2 transactivation domain of human papillomavirus type 11 (PDB entry 1r6n; Wang *et al.*, 2004 ▶; Yoakim *et al.*, 2003 ▶; White *et al.*, 2003 ▶). In the latter case, inhibitors were shown to antagonize the interaction of E2 with E1 helicase. The occurrence of the two 3′,5′-ADP molecules in the AcpS_BA_ structure may be a clue to the design of inhibitors that may prevent the binding of CoA. Their affinity might be comparable to that of CoA primarily based on similar binding of ADP_I_ and the nucleotide part of CoA in AcpS_VC_, AcpS_BS_ and AcpS_SC_. Superposition of the AcpS_BA_ structure (PDB entry 3hyk) with the AcpS_VC_ structure (PDB entry 3qmn) has revealed further interesting details that could be useful in the design of inhibitors. For example, the P atom of the 5′-β-phosphate of CoA/K (Fig. 3 ▶ *c*) is only 2.74 and 1.33 Å from the P atom of the 3′-P of ADP_II_ (Fig. 4 ▶ *c*) and Mg-III (Fig. 4 ▶ *f*), respectively. Moreover, the conserved Arg28 that is known to be crucial for ACP–AcpS binding (Parris *et al.*, 2000 ▶) interacts with ADP_II_ in the AcpS_BA_ structure (Fig. 4 ▶ *c*) and with the P-­pant moiety of some CoAs in AcpS_VC_ and other available CoA–AcpS complex structures. Thus, similar contacts between the P-pant group of CoA with AcpS_VC_ and AcpS_BS_ and between ADP_II_ with AcpS_BA_ may strengthen the binding of potential CoA inhibitors. Although the unusual presence of ADP_II_ depends in part on lattice packing and the high concentration of magnesium (Figs. 4 ▶ *d*, 4 ▶ *e* and 4 ▶ *f*), it could also block and/or decrease the affinity of ACP–AcpS interaction (Fig. 6 ▶). Superposition of the AcpS_BA_ and ACP_BS_–AcpS_BS_ complex (PDB entry 1f80) structures has shown that ADP_II_ clashes with the predicted positions of residues Gly33–Glu41 in ACP_BS_ (Fig. 6 ▶). This region contains the active Ser36. There is a resemblance between the ADP_II_ O4′–AcpS_BA_ Arg14 interaction (Fig. 4 ▶ *c*) and the ACP_BS_ Asp35/Asp38–AcpS_BS_ Arg14 interaction, with the latter being important for the correct orientation of Ser36 of the carrier protein in the P-­pant transfer reaction (Parris *et al.*, 2000 ▶). The rest of the ADP_II_ atoms do not mimic any of the ACP_BS_–AcpS_BS_ contacts, but instead interact with Lys70 and Glu71 of the symmetry-related chain *C* and Arg84 of the symmetry-related chain *B*. Hydrophobic interactions play a predominant role in the formation of a complex between human AcpS and its cognate ACP, whereas ionic forces stabilize complexes between the bacterial homologs (Bunkoczi *et al.*, 2007 ▶). Therefore, a bulky hydrophilic ligand similar to the two 3′,5′-ADP molecules may specifically target bacterial AcpS (Fig. 7 ▶). Lastly, it is important to stress the specific ADP_I_ 5′-P–ADP_II_ 3′-P interaction through Mg-III. The preference for the observed ADP_I_ 5′-P–Mg-III–ADP_II_ 3′-P interaction as opposed to an ADP_I_ 5′-P–Mg-III–ADP_II_ 5′-P interaction is most likely to reflect the energy minimum for both 3′,5′-ADPs to interact in the protein crystal lattice. Although we think that ADP_II_ is a crystallographic artifact, the biological relevance of ADP_II_ should be further investigated.

## 5. Conclusions

The importance of FAS II structural studies for the development of effective therapeutics continues to stimulate research to discover and characterize homologous FAS II enzymes from a variety of human pathogens. AcpS is an important enzyme in regulation of the FAS II pathway. This protein triggers FAS II by post-translationally modifying ACP. We have determined three structures of the AcpS enzymes from three different human pathogens. Consistent with previously published data, our results also imply that these enzymes do not significantly change their tertiary structures upon binding substrates. A limiting factor in the production of the desired ACP intermediates in FAS II seems to be the quaternary architecture of AcpS. The high concentration of magnesium in the crystallization condition resulted in the hydrolysis of CoA by AcpS_BA_ even in the absence of ACP. The unusual binding of ADP_II_ in AcpS_BA_ suggests a strategy for the inhibition of bacterial AcpS. For example, the presence of a bulky ligand that would mimic the two bound 3′,5′-ADP molecules at the interaction interface between two adjacent monomers in the AcpS trimer may prevent the binding of CoA and/or the formation of a productive CoA–AcpS–ACP complex.

## Supplementary Material

PDB reference: *Staphylococcus aureus* AcpS, 3f09

PDB reference: *Bacillus anthracis* AcpS, 3hyk

PDB reference: *Vibrio cholerae* AcpS, 3qmn

Supplementary material file. DOI: 10.1107/S0907444912029101/dw5015sup1.pdf

Enhanced figure: interactive version of Fig. 7

## Figures and Tables

**Figure 5:**
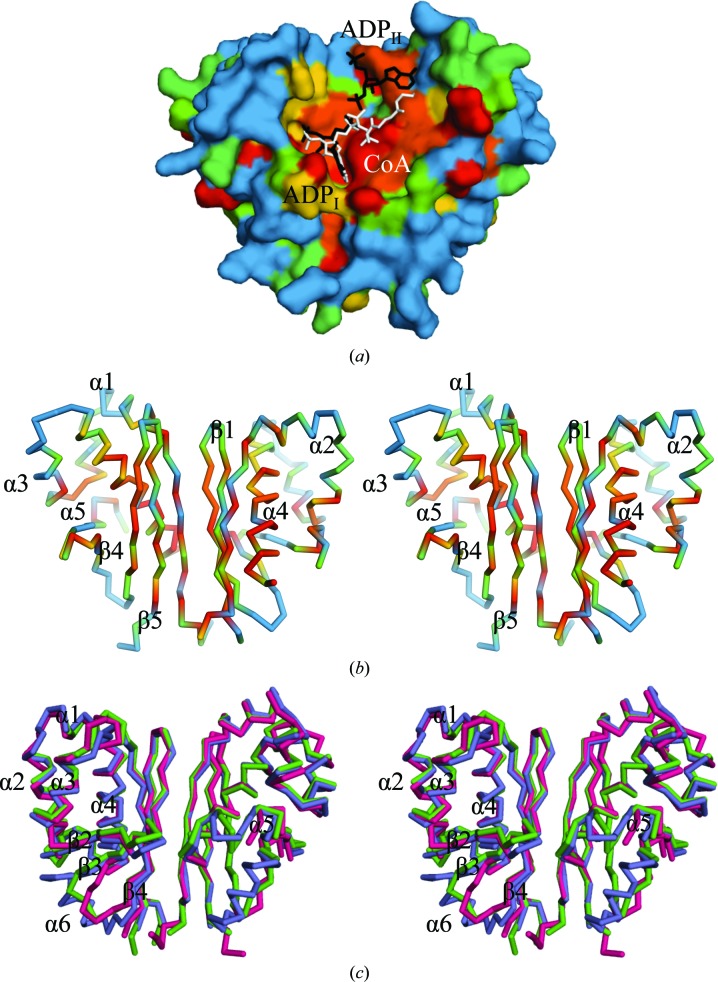
Structural comparison of bacterial AcpS. (*a*) Surface diagram of the sequence-based structural alignment of the bacterial AcpS enzymes presented in Fig. 1 ▶(*a*). The structure of apo-AcpS_SA_ is used to display the conservation of the residues, which are colored from blue (variable) to red (100% conserved). The positions of two 3′,5′-ADP molecules (black) in AcpS_BA_ and of CoA (white) in AcpS_VC_ are shown. (*b*) Stereogram of the monomer–monomer interaction interface in the AcpS_SA_ trimer. Residues are colored from blue (variable) to red (100% conserved). The third molecule is omitted for clarity. (*c*) Stereogram of the least-squares superposition of the AcpS_SA_ (red), AcpS_VC_ (blue) and AcpS_BA_ (green) structures.

**Table 1:** Data-collection and refinement statistics Values in parentheses are for the highest resolution shell. na, not applicable.

	AcpS_SA_	AcpS_VC_	AcpS_BA_
Data collection			
Wavelength (Å)	0.9787	0.9794	0.9787
Data-collection temperature (K)	100	100	100
Space group	*P*2_1_2_1_2_1_	*P*2_1_	*P*3_2_21
Unit-cell parameters (Å, °)	*a* = 67.39, *b* = 77.4, *c* = 81.5	*a* = 94.55, *b* = 139.04, *c* = 138.32, β = 93.71	*a* = *b* = 77.53, *c* = 122.96, γ = 120.0
Resolution range (Å)	30.00–1.82 (1.86–1.82)	45.55–1.85 (1.88–1.85)	34.99–2.31 (2.35–2.31)
No. of reflections	37963	300465	19381
*R*_merge_ (%)	5.1 (54.3)	9.1 (54.0)	12.3 (55.3)
Completeness (%)	98.3 (91.8)	99.4 (99.7)	99.9 (100.0)
〈*I*/σ(*I*)〉	24.97 (2.58)	24.2 (2.00)	20.54 (4.90)
Multiplicity	5.5 (4.9)	3.7 (3.7)	10.2 (9.9)
Wilson *B* factor (Å^2^)	29.9	23.4	30.2
Refinement			
Resolution range (Å)	30.00–1.82 (1.87–1.82)	29.89–1.85 (1.90–1.85)	34.99–2.31 (2.37–2.31)
No. of reflections	36019 (2432)	284976 (20744)	18306 (1299)
*R*_work_/*R*_free_ (%)	18.94/23.22	15.61/19.13	18.96/24.65
Protein molecules/atoms	3/3471	24/23277	3/2811
Substrate/product	na/na	CoA/3′,5′-ADP	na/3′,5′-ADP
Solvent atoms	314	2487	266
Mean temperature factor (Å^2^)	16.27	27.61	16.96
Coordinate deviation			
R.m.s.d. bonds (Å )	0.013	0.012	0.011
R.m.s.d. angles (°)	1.404	1.755	1.744
Ramachandran plot†			
Most favored (%)	92.2	94.0	96.8
Allowed (%)	7.1	6.0	3.2
Generously allowed (%)	0.6	0.0	0.0
Outside allowed (%)	0.0	0.0	0.0

† Statistics are based on *PROCHECK* (Laskowski *et al.*, 1993 ▶)

## Footnotes

1 Supplementary material has been deposited in the IUCr electronic archive (Reference: DW5015). Services for accessing this material are described at the back of the journal.
