# Supplementary material for: Structural characterization and comparison of three acyl-carrier-protein synthases from pathogenic bacteria
Source: Acta Crystallogr D Biol Crystallogr. 2012 Sep 13;68(Pt 10):1359–70. doi: 10.1107/S0907444912029101 (PMC3447402; doi:10.1107/S0907444912029101)
Supplement: Supplementary file 1 [file d-68-01359-sup1.pdf]

Supplementary Material

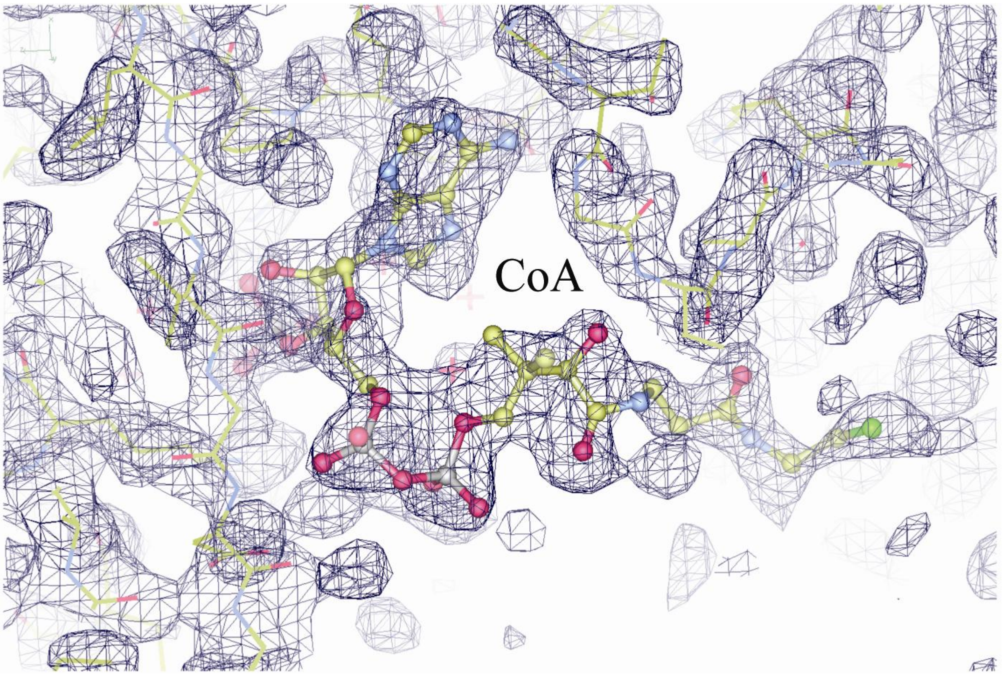

Supplementary Figure 1. Experimental density map (AcpS$_{VC}$ structure) after density modification countered at 1.28σ.
